# Safe Listening Beliefs, Attitudes, and Practices Among Gamers and Esports Participants: International Web-Based Survey

**DOI:** 10.2196/60476

**Published:** 2025-03-25

**Authors:** Nicola Diviani, Shelly Chadha, Peter Mulas, Sara Rubinelli

**Affiliations:** 1 Swiss Paraplegic Research Nottwil Switzerland; 2 Faculty of Health Sciences and Medicine University of Lucerne Lucerne Switzerland; 3 Center for Rehabilitation in Global Health Systems University of Lucerne Lucerne Switzerland; 4 Department of Noncommunicable Diseases World Health Organization Geneva Switzerland

**Keywords:** video gaming, esports, hearing health, safe listening, auditory risk, health behavior, intervention design, games, listening, auditory, survey, college, data analysis, awareness, listening behavior, gaming

## Abstract

**Background:**

The global rise of video gaming and esports has raised significant concerns about hearing loss due to loud sound exposure. While these activities provide entertainment and have applications in health care, the auditory health risks and behavioral factors influencing listening habits among gamers remain underexplored. Research is needed to develop tailored interventions that address the unique barriers, attitudes, and beliefs of gamers and esports participants, promoting safer listening practices and minimizing auditory health risks.

**Objective:**

This study aimed to explore listening behaviors, attitudes, and awareness regarding hearing health risks among video gamers and esports participants. The findings are intended to guide the design and implementation of technological features that encourage safer listening practices, in alignment with the World Health Organization’s Safe Listening initiative.

**Methods:**

An open web-based survey was conducted from September 2022 to January 2023, targeting video gamers and esports enthusiasts. Participants were recruited via World Health Organization social media platforms and outreach to stakeholders. The survey assessed gaming behaviors, listening habits, awareness about hearing health, beliefs, readiness to change listening behaviors, and communication preferences. Data were analyzed using descriptive statistics and multinomial logistic regression.

**Results:**

A total of 488 responses were collected, with 67.2% (n=328) of participants identifying as male, and 56.4% (n=275) having a college degree or higher. Of the respondents, 90.8% (n=443) were actively engaged in video gaming, while 54.9% (n=268) viewed esports, and 13.9% (n=68) participated in esports events. Notably, 24.8% (n=110) of gamers, 18.3% (n=49) of esports viewers, and 37.1% (n=23) of esports players reported using high or very high volume settings. Despite around half of the participants experiencing symptoms indicative of hearing damage (eg, ringing in the ears), only 34.3% (n=152) of gamers, 35.8% (n=92) of esports players, and 39.7% (n=27) of esports viewers reported taking sound breaks every hour. The study identified a balanced distribution across readiness-to-change stages, with 30.3% (n=148) in the precontemplation stage, 35.3% (n=173) in the contemplation stage, and 34.2% (n=167) in the action stage. Factors such as perceived susceptibility to hearing loss, perceived benefits of preventive action, and self-efficacy significantly influenced readiness to change. Communication preferences indicated that 51% (n=249) of participants were interested in receiving more information on hearing health, with health care professionals and governmental agencies being the most trusted sources.

**Conclusions:**

The findings highlight an urgent need for interventions to promote safe listening practices among gamers, emphasizing a gap between awareness and preventive action. The integration of safe listening features into video games and esports platforms, along with targeted communication strategies, can enhance auditory health awareness and protective behaviors. Future research should evaluate the effectiveness of these interventions to ensure comprehensive auditory health protection in the digital entertainment sector.

## Introduction

Video gaming and esports have undergone an exponential rise in recent years, evolving into a major entertainment force that captivates individuals across different age groups [1]. With projections suggesting that the global video game market will grow at an annual rate of 12.9% from 2022 to 2030—reaching an estimated US $399.6 billion by 2028—and the global gamer population increasing from 2.7 billion in 2021 to an expected 3.04 billion by 2027, the influence of these digital platforms is significant [2,3]. Concurrently, esports has experienced a surge in popularity, with its global audience forecasted to reach 577 million by 2025, and the market is anticipated to grow at a rate of 16.7% annually, resulting in a value of US $4.28 billion by 2030 [4,5].

Beyond serving as engaging and entertaining mediums, video games and esports are increasingly acknowledged for their potential in health care, particularly through serious gaming applications aimed at education, rehabilitation, and therapy, thus highlighting their versatile utility beyond mere entertainment [6-8]. Despite these benefits, concerns about the potential health risks associated with video gaming and esports have emerged. The documented impacts on mental and physical health are compounded by the risks of hearing loss, tinnitus, and other auditory disorders resulting from exposure to loud and persistent sound effects inherent in these activities [9-11]. Echoing the World Health Organization’s (WHO) warning, over 1 billion young people are at an elevated risk of hearing loss due to recreational exposure to loud sounds, including the use of personal audio devices and attendance at loud public events [12].

The public health realm has seen significant attention directed toward the issue of hearing loss from recreational sound exposure, leading to a wealth of research and the development of global standards aimed at mitigating this risk. The pervasive nature of this issue, covering activities ranging from personal listening device use to attendance at live events with high decibel levels, has been illuminated through extensive research. Such studies have consistently underscored the risk of hearing damage from prolonged exposure to loud music via earphones or headphones and attendance at entertainment venues where volumes exceed safe listening thresholds, potentially leading to noise-induced hearing loss [13-16].

In response, the WHO and the International Telecommunication Union have collaborated to launch the “Make Listening Safe” initiative, which recommends standards for incorporating safe listening features into personal audio devices. This includes software that monitors volume levels and exposure time, aiming to regulate sound exposure in recreational settings and promote a balance between enjoyment and health [17,18]. These standards not only aim to raise awareness among the public and stakeholders about the risks associated with loud sound exposure but also to provide concrete guidelines for safe listening practices, such as sound level limits and user warnings about unsafe listening volumes.

Research focused on hearing health within the context of video gaming and esports, on the other hand, remains practically nonexistent, particularly concerning unsafe listening habits and their contributing factors [19,20]. Existing research from different fields, such as physical activity promotion [21], and related areas like safe listening using personal audio devices [13,14], as well as behavioral models such as the health belief model (HBM) [22], can be useful starting points to better understand the factors influencing behavior and the barriers that need to be addressed. However, we know that interventions aimed at changing health behavior must be tailored to the specific characteristics and preferences of the population being targeted. A factor that plays a significant role in one context might indeed not have the same impact in another. For example, self-efficacy may be a major determinant for behaviors requiring significant skill, such as learning new physical activities [23], but may be less relevant for behaviors like adjusting volume settings, which require minimal effort. Similarly, barriers to safe listening, such as the worry about not enjoying music in noisy environments, could be a major factor for users of personal audio devices outdoors [13] but not for gamers who play indoors and often in solitude. In the gaming context, other factors, such as immersion or competitive performance, may be more significant barriers. The large body of existing evidence from other fields may therefore not directly apply to the gaming and esports environments, as behavioral models used to understand determinants of behavior must be specifically adapted to this context. Only by understanding how these models apply to video gamers and esports participants, we can effectively tailor interventions and communication strategies.

Our study specifically aims to address this gap by (1) gaining insights into the listening habits and safe listening behaviors of video gamers and esports players, exploring the determinants of these behaviors; (2) understanding the link between attitudes, beliefs, awareness, and stages of change regarding auditory health; and (3) identifying the most effective sources of information that can be leveraged to promote safer listening practices. These findings will support the development of interventions that can be integrated into industry standards, contributing to the design of technological features that encourage safer listening within gaming and esports platforms. By aligning with the WHO Safe Listening initiative, our work aims to ensure that auditory health considerations are integrated into the rapidly evolving landscape of video gaming and esports, fostering a more holistic approach to hearing health across all recreational sound exposure settings.

## Methods

### Sample

From September 2022 to January 2023, an open, web-based survey was shared on the WHO’s social media platforms, including posts on X (formerly Twitter) and Facebook. These posts were designed to reach a broad convenience sample of video gamers and esports enthusiasts. Additionally, key stakeholders were contacted via email and encouraged to share the survey link within their local networks to increase participation. No paid advertising or boosted posts were used for recruitment. Participation in the survey was completely voluntary, and no incentives or compensation were offered to participants. To participate in the survey, individuals had to have a good command of English, Spanish, French, or Chinese and be either video game players or involved in esports. No further specific eligibility criteria for access were specified, and participants could freely choose to participate. Of the 1043 individuals who submitted the first survey page, 532 (51%) completed the survey and were retained to be included in the analyses. To ensure that participants responded to the survey with adequate attention, response time was evaluated, and 35 (6.7%) individuals who completed the survey in less than 5 minutes were excluded, resulting in a total of 488 responses retained for the analyses.

### Instrument and Measures

#### Overview

To develop the web-based questionnaire, a combination of established theories and empirical studies was used. Specifically, the HMB [22], the transtheoretical model (TTM) of change [24], and previous research on music- and noise-induced hearing loss were drawn upon [25]. The HBM suggests that individuals are more likely to adopt preventive measures if they perceive themselves to be at risk of developing a severe health condition (perceived susceptibility and severity), believe that taking action can prevent it (perceived benefits), and have the confidence to perform the necessary actions (self-efficacy). The TTM suggests that individuals can be categorized into different groups based on their readiness to adopt preventive behaviors.

The questionnaire (Multimedia Appendix 1) consisted of seven main sections designed to assess various aspects of participants’ listening habits when gaming or engaging in esports. These sections included questions on (1) their gaming or esports behaviors and related listening habits (respondents were redirected to different sections of the survey depending on whether they played videogames, watched esports, participated in esports, or a combination), (2) the consequences of their listening behavior, (3) their knowledge and awareness of hearing loss, (4) their beliefs about hearing loss and safe listening, (5) their readiness to change their listening behavior, (6) their desire for information on hearing loss and safe listening and their preferred sources of information, and (7) their sociodemographic information.

The survey consisted of 10 pages, each containing 3 to 7 items. Respondents were required to answer basic and sociodemographic questions, which included a nonresponse option. All other items were optional and could be left unanswered. Completeness checks were performed after submission, highlighting mandatory items if incomplete. A “Back” button allowed respondents to review and modify their answers before final submission.

The survey was pretested with 15 individuals who had experience in video gaming or esports. Their feedback helped refine the questions for clarity and relevance, ensuring that the final version was suitable for the target population. The survey was hosted on the Qualtrics web-based platform, a widely used platform that ensures data security through end-to-end encryption and complies with General Data Protection Regulation guidelines to protect participant anonymity. Several accessibility features, such as adjustable text sizes and screen reader compatibility, were included to accommodate participants with disabilities.

To prevent multiple submissions from the same participant, cookies were used on the introduction page and were valid for 24 months. IP address checks were not used to identify duplicate entries. Data collected through the survey were stored in secure cloud storage provided by Qualtrics, with all responses anonymized, and participants assigned unique identifiers to ensure confidentiality. The data will be securely stored for 5 years and then deleted in compliance with data protection regulations. We adhered to the CHERRIES (Checklist for Reporting Results of Internet E-Surveys; Multimedia Appendix 2) for reporting the results.

#### Gaming and Esports Behaviors and Related Listening Habits

To assess the frequency of video game or esports viewing or playing and volume-level preferences, a series of single-item indicators were used. Participants were asked to rate the frequency of their gaming or esports activities, as well as the volume level they typically set on their devices, using a scale ranging from 1=minimum volume to 10=maximum volume. These questions were adapted from measures used in prior research on the topic, such as the Listening Habits Questionnaire (LHQ) [25].

#### Preventive Behaviors and Consequences of Listening

Participants were asked to indicate whether they take a break from sound every hour and whether they check the sound dose. The frequency of experiencing a ringing or fuzziness in the ears after playing video games or engaging in esports was measured on scales ranging from 1=never to 5=always. Participants were asked to rate how often they experienced these symptoms using questions from the LHQ [25].

#### Awareness and Knowledge

Participants were presented with 3 statements related to hearing loss in the context of gaming and esports, which were collaboratively developed with a team of international experts in the field of hearing. The statements included “listening to sounds above 85dB over a period of time can cause permanent damage to your hearing,” “the amount of time you listen to a sound affects how much damage it will cause,” and “listening to video games or esports sounds at loud listening levels may damage hearing.” Participants were asked to indicate whether they believed each statement to be true or false and were scored accordingly. A knowledge score was then computed by summing the scores for all 3 statements, resulting in a range of 0 to 3. The average knowledge score was 2.77 (SD 0.61), with an α coefficient of .690.

#### Beliefs

An adapted version of a section of the LHQ [25] was used to assess the constructs of the HBM. The adaptation involved removing the distinction between quiet and loud environments after internal pretesting. Participants were then asked to rate their agreement on a 7-point scale (with higher scores indicating greater agreement) on a total of 22 statements about hearing loss susceptibility related to gaming and esports. These statements covered perceived susceptibility to hearing loss (4 items, α=.776), perceived severity of hearing loss (6 items, α=.865), benefits of preventing hearing loss (5 items, α=.830), barriers to preventing hearing loss (3 items, α=.638), and self-efficacy for taking preventative action (4 items, α=.845). The scores for each construct were obtained by averaging the individual scores on items within each subscale.

#### Readiness to Change

To assess readiness to change listening behaviors, a set of 12 statements was adapted from a survey by Rollnick et al [26] for excessive drinkers and a previous study on safe listening in the context of personal listening devices [13]. Each statement represented one aspect of the 3 stages of the TTM (precontemplation, contemplation, and action). Respondents answered each statement on a 5-point scale (ranging from 2=strongly disagree to +2=strongly agree), as per the authors’ instructions. The subscales showed acceptable to good internal consistency (precontemplation: α=.626; contemplation: α=.740; and action: α=.784). Scores for items related to the same stage were added, resulting in a stage score ranging from –8 to +8. The respondent’s stage of change was determined by the stage with the highest score. If 2 stages had equally high scores, the stage furthest along the change process was chosen (eg, if precontemplation and contemplation had the highest scores, contemplation was designated as the stage of change).

#### Communication Needs and Preferences

Participants were asked if they desired more information about hearing loss and safe listening, with response options of yes or no. Those who answered affirmatively were then asked to indicate their preferred channels for receiving this information on a 7-point scale, ranging from 1=definitely no to 7=definitely yes, which included options such as public health campaigns, gaming device interfaces, and social media. Additionally, all participants were asked to rate how much they trust in different sources of information about hearing loss and safe listening, such as health care professionals, governmental and international agencies, and family and friends, using a 7-point scale ranging from 1=not at all to 7=completely.

#### Sociodemographics

Participants were requested to provide information about their sex, age, the highest level of education completed, and country of residence. For analytical purposes, sex (male and female), age (16-35 and >35 years), and educational level (low and high) were dichotomized.

### Data Analysis

The data were analyzed using SPSS Statistics (version 21.0; IBM Corp). Descriptive statistics, including basic frequencies, were calculated to provide an overview of the sample characteristics across all variables of interest, such as demographics, knowledge, and beliefs related to hearing health. To assess group differences in the descriptive analyses, we used chi-square tests for categorical variables and 1-way ANOVAs for continuous variables, supplemented by effect size measures such as Cramer *V* or ϕ for chi-square tests and partial eta squared (η_p_^2^) for ANOVAs. These tests and effect size measures allowed us to compare key differences between sex, age, and education subgroups within the sample, providing a more detailed understanding of both the statistical significance and the magnitude of group differences across the variables of interest. To assess the relationship between predictors (knowledge and beliefs about hearing health) and the outcome variable (readiness to change listening behaviors), we performed a multinomial logistic regression analysis. We chose this method because the outcome variable (readiness to change) is categorical with multiple levels, and it allowed us to evaluate how each predictor influenced the likelihood of participants being in different stages of readiness to change while controlling for other variables. The use of multinomial logistic regression was particularly suited to account for the complexity of the data and to handle multiple predictors influencing a nonbinary categorical outcome. All assumptions for the chi-square tests, ANOVAs, and multinomial logistic regression (such as normality, independence, and multicollinearity) were tested and met, ensuring the robustness of our findings.

### Ethical Considerations

Ethics approval for research in Switzerland is governed by the Federal Act on Research Involving Human Beings (Human Research Act, HRA, SR 810.30 [27]). According to chapter 1, article 2, paragraph 2 of this act, ethics approval is required for research involving human participants. However, our study, which involves an anonymous web-based survey, does not fall within the scope of the HRA. Specifically, article 2, paragraph 2 states: “It [the HRA] does not apply to research which involves: a. IVF embryos in accordance with the Stem Cell Research Act of 19 December 2003; b. anonymized biological material; c. anonymously collected or anonymized health-related data.” Therefore, our project does not require ethics approval (see also the Swissethics decision tree [28]). This web-based anonymous survey was conducted in strict adherence to the principles outlined in the Declaration of Helsinki. Informed consent was obtained from all participants prior to starting the survey. Participants were presented with a web-based informed consent form, which included information about the voluntary nature of the study, data anonymity, and their right to withdraw at any time without any consequences. Consent was confirmed through a mandatory checkbox before proceeding to the survey questions. No compensation was provided to study participants.

## Results

### Overview

Of the survey participants, the majority were male (n=355, 67.7%), with an average age of 28.4 (SD 10.8) years. The highest level of education attained by most respondents was a 4-year college degree (n=107, 21.9%) or a high school diploma or General Educational Development (n=101, 20.7%). Although respondents hailed from 92 different countries, the largest number of respondents came from the United States (n=70, 14.3%), followed by the United Kingdom (n=51, 10.5%) and India (n=40, 8.2%). More details about the participants are shown in Table 1.

**Table 1:** Sample characteristics (N=488).

		Values, n (%)
**Sex**		
	Male	328 (67.2)
	Female	144 (29.5)
	No answer	16 (3.3)
**Age (years)**		
	16-35	386 (79.1)
	>35	102 (20.9)
**Education**		
	Low education (less than college)	196 (40.2)
	Middle education (college)	133 (27.3)
	High education (more than college)	142 (29.1)
	No answer	17 (3.5)
**Country of origin (top 5)**		
	United States	70 (14.3)
	United Kingdom	51 (10.5)
	India	40 (8.2)
	Belgium	34 (7)
	Australia	21 (4.3)
**Involved in** **(more than 1 answer possible)**		
	Video games	443 (90.8)
	Esports viewing	268 (54.9)
	Esports participation	68 (13.9)

### Gaming and Esports Behaviors and Related Listening Habits

Details of the overall gaming and esports behaviors and the related listening habits are shown in Table 2 (video game players) and Table 3 (esports viewers and esports players).

**Table 2:** Behaviors and related listening habits of video game players (n=443).

		Values
**Time spent playing (hours per week), n (%)**		
	0-1	29 (6.5)
	1-2	30 (6.8)
	3-4	50 (11.3)
	4-6	49 (11.1)
	6-10	73 (16.5)
	10-15	74 (16.7)
	15-30	82 (18.5)
	>30	56 (12.6)
Volume setting, mean (SD) (scale 1-100)		47.6 (21.3)
**Volume setting, n (%)**		
	Very low (0%-20%)	49 (11.1)
	Low (20%-40%)	134 (30.2)
	Medium (40%-60%)	150 (33.9)
	High (60%-80%)	85 (19.2)
	Very high (80%-100%)	25 (5.6)
**Importance of sounds, n (%)**		
	Not at all important	14 (3.2)
	Not so important	25 (5.6)
	Somewhat important	87 (19.6)
	Very important	151 (34.1)
	Extremely important	166 (37.5)
**Sound break every hour, n (%)**		
	Yes	152 (34.3)
**Checks use statistics, n (%)**		
	Yes	45 (10.2)
**Experience ringing in ears, n (%)**		
	Never	253 (57.1)
	Rarely	125 (28.2)
	Sometimes	51 (11.5)
	Often	6 (1.4)
	Always	8 (1.8)
**Experience fuzziness in ears, n (%)**		
	Never	206 (46.5)
	Rarely	148 (33.4)
	Sometimes	71 (16)
	Often	11 (2.5)
	Always	7 (1.6)

**Table 3:** Behaviors and related listening habits of esports viewers and players.

			Viewers (n=287)		Players (n=62)
**Frequency of viewing, n (%)**					
	Daily	14 (5.2)		15 (24.2)	
	Weekly	75 (28)		23 (37.1)	
	Monthly	79 (29.5)		11 (17.7)	
	Yearly	68 (25.4)		6 (9.7)	
	Less than yearly	32 (11.9)		7 (11.3)	
Volume setting, mean (SD) (scale 1-100)			45.6 (20.4)		55.7 (22.5)
**Volume setting, n (%)**					
	Very low (0%-20%)	34 (12.7)		5 (8.1)	
	Low (20%-40%)	80 (29.9)		10 (16.1)	
	Medium (40%-60%)	105 (39.2)		24 (38.7)	
	High (60%-80%)	36 (13.4)		14 (22.6)	
	Very high (80%-100%)	13 (4.9)		9 (14.5)	
**Experience ringing in ears, n (%)**					
	Never	186 (69.4)		36 (53.7)	
	Rarely	46 (17.2)		20 (29.9)	
	Sometimes	29 (10.8)		6 (9)	
	Often	2 (0.7)		0 (0)	
	Always	5 (1.9)		5 (7.5)	
**Experience fuzziness in ears, n (%)**					
	Never	187 (69.8)		33 (49.3)	
	Rarely	49 (18.3)		18 (26.9)	
	Sometimes	23 (8.6)		8 (11.9)	
	Often	4 (1.5)		3 (4.5)	
	Always	5 (1.9)		5 (7.5)	
**Importance of sounds, n (%)**					
	Not at all important	10 (3.7)		1 (1.5)	
	Not so important	33 (12.3)		8 (11.8)	
	Somewhat important	79 (29.5)		11 (16.2)	
	Very important	80 (29.9)		20 (29.4)	
	Extremely important	66 (24.6)		28 (41.2)	
**Break from sound every hour, n (%)**					
	Yes	96 (35.8)		27 (39.7)	
**Checks use statistics, n (%)**					
	Yes	37 (13.8)		12 (17.6)	

#### Video Game Players

Of the surveyed participants, 90.8% (n=443) reported engaging in video games (Table 2). Around one-third of gamers (n=158, 35.7%) reported playing for up to 6 hours per week, followed by a second group (n=147, 33.2%) who played between 6 and 15 hours per week, and a third group (n=138, 31.1%) who played more than 15 hours per week. Male respondents reported significantly more time spent on video gaming compared to female respondents (*P*=.008; ϕ=0.210), while those with a higher educational level spent significantly less time playing video games compared to those in the lower and medium educational groups (*P*=.03; Cramer *V*=0.175). The average volume setting reported by participants was just below 50% (mean 47.6, SD 21.3), with almost 1 in 4 respondents listening at a high or very high volume (n=110, 24.8%). No significant differences were found across sexes, ages, or educational levels. Finally, most respondents indicated that sounds are either extremely (n=166, 37.5%) or very (n=151, 34.1%) important when playing video games. Male respondents, on average, placed greater importance on video game sounds than female respondents (*P*=.03; Cramer *V*=0.161).

#### Esports Viewers and Players

More than half of the study participants (n=281, 57.6%) reported engaging in esports, with most of them only viewing (n=213, 75.8%) and approximately one-fifth (n=55, 19.6%) both viewing and participating (Table 3). A small percentage (n=13, 4.6%) reported only participation. Among viewers (n=268), the majority viewed on a monthly basis (n=79, 29.5%). Male participants were found to watch esports events more often than female participants (*P*=.03; Cramer *V*=0.202). The average volume setting reported was slightly below 50% (mean 45.6, SD 20.4), with almost 1 in 5 respondents listening at a high or very high volume (n=49, 18.3%). Participants with lower educational levels reported significantly higher volume settings when watching esports events (*F*_2,254_=3.139; *P*=.04), while no significant differences were observed across sex or age groups.

Among esports participants (n=68), the majority took part in esports events on a weekly basis (n=23, 37.1%). No significant differences were observed regarding sex, age, or education. The average volume setting for esports participation was higher than for viewing at 55.7 (SD 22.5), with more than 1 in 5 respondents listening at a high or very high volume (n=23, 21%). No significant differences were observed across sexes, ages, educational levels, or frequency of participation.

Overall, esports viewers and players consider sounds in esports as either somewhat (n=82, 29.2%), very (n=85, 30.2%), or extremely (n=68, 24.2%) important. Among esports players, in particular, sound was considered extremely important by a majority (n=28, 41.2%). Participants with lower educational levels placed significantly greater importance on sounds in esports events compared to those with higher educational levels (*P*=.02; Cramer *V*=0.183).

### Protective Behavior and Consequences of Listening

Only about one-third of video game players (n=152, 34.3%) reported taking a break from sound every hour. Just 1 in 10 (n=45, 10.2%) reported checking the sound dosage information provided by the gaming device. Male and female respondents differed significantly in whether they checked information on how much time they spend listening and their sound dosages through their gaming device (*P*=.03; Cramer *V*=0.144). A similar picture emerged for esports players and participants. Over one-third of them (n=106, 37.7%) reported taking an hourly break, and only a few reported checking the sound dosage (n=43, 15.3%). No group differences were observed.

Regarding the consequences of listening, almost half of the video game players reported having experienced at least once a ringing in their ears after playing (n=190, 42.9%), and more than half have experienced fullness or fuzziness in their ears (n=237, 53.5%). Female respondents reported feeling their ears full or fuzzy after playing video games more often than male respondents (*P*=.006; Cramer *V*=0.184). Regarding esports, we observed 2 different pictures for viewers and players. Among esports viewers, less than one-third reported having experienced a ringing in their ears (n=82, 30.6%) or a feeling of fullness or fuzziness (n=81, 30.2%) after watching esports. Among esports players, however, this percentage increased to 46.3% (n=31) for the ringing in their ears and to 50.7% (n=34) for the feeling of fullness or fuzziness. Participants’ characteristics were not associated with the frequency of experiencing consequences.

### Awareness and Knowledge

Overall, the respondents were very knowledgeable about the risks for hearing related to exposure to video games, scoring on average 2.78 (SD 0.62) of a maximum of 3 points indicating a correct answer to all questions. No significant differences among groups were observed.

### Beliefs

The majority of participants rated the perceived severity of hearing loss as high (mean 5.84, SD 1.31), while their perceived susceptibility to it was relatively low (mean 3.70, SD 1.54). Participants recognized the benefits of preventing hearing loss (mean 5.84, SD 1.22) but also faced significant barriers to doing so (mean 4.53, SD 1.56), such as the belief that turning down the volume would limit their enjoyment of video games and esports. Despite this, respondents demonstrated high confidence in their ability to modify their listening behavior (mean 5.24, SD 1.57). We observed few group differences in beliefs, except for higher self-efficacy among male respondents (*F*_1,470_=4.13; *P*=.04; η_p_^2^=0.009) and lower perceived susceptibility among younger respondents (*F*_1,486_=12.33; *P*<.001; η_p_^2^=0.025). In addition, respondents with higher education reported perceiving more benefits of prevention compared to the other 2 groups (*F*_2,468_=3.65; *P*=.03; η_p_^2^=0.015).

### Readiness to Change

#### Overview

We observed a balanced distribution of participants into the 3 readiness-to-change groups. Most of them (n=173, 35.3%) could be categorized as belonging to the “contemplation” stage, followed by the “action” stage (n=167, 34.2%) and the “precontemplation” stage (n=148, 30.3%). A chi-square test revealed a significant association between educational level and stage of change (*P*=.009; Cramer *V*=0.120), with individuals in the high education group more likely to be in the action stage (n=60, 42.3%) compared to those in the medium (n=50, 37.6%) and low education groups (n=49, 25%). Table 4 shows the multinomial logistic regression results for factors associated with being in one of the 3 readiness-to-change stages. Those who were in the contemplation stage were the reference category.

**Table 4:** Associations of knowledge and beliefs with readiness to change (N=488)^a^.

	Readiness to change				
	Precontemplation			Action	
	OR^b^ (95% CI)	*P* value	OR (95% CI)		*P* value
Knowledge	1.293 (0.769-0.174)	.33	0.998 (0.628-1.584)		.99
Perceived susceptibility	0.557 (0.452-0.687)	<.001	1.031 (0.851-1.249)		.76
Perceived severity	1.344 (1.056-1.712)	.02	1.082 (0.868-1.349)		.48
Benefits of preventive action	0.611 (0.461-0.809)	.001	0.968 (0.732-1.281)		.82
Barriers to preventive action	0.985 (0.818-1.186)	.54	0.808 (0.680-0.959)		.01
Self-efficacy in prevention	1.518 (1.237-1.863)	<.001	1.431 (1.196-1.711)		<.001

^a^Multinomial logistic regression analysis with contemplation as the reference category. The model is adjusted for sex, age, and education. Pseudo *R*^2^: Cox and Snell=0.274; Nagelkerke=0.308.

^b^OR: odds ratio.

#### Multivariate Results: Contemplation Versus Precontemplation Stage

The second column of Table 4 shows the odds ratio (OR) for being in the precontemplation stage versus being in the contemplation stage for each variable listed. The odds of being in the precontemplation versus the contemplation group decrease the more one perceives to be susceptible to hearing loss (OR 0.557, 95% CI 0.452-0.687) and the more one perceives the benefits of preventive actions (OR 0.611, 95% CI 0.461-0.809). On the other hand, the odds of being in the precontemplation group increase the more one perceives hearing loss as severe (OR 1.344, 95% CI 1.056-1.712) and the more one has self-efficacy in the prevention of hearing loss (OR 1.518, 95% CI 1.237-1.863).

#### Multivariate Results: Contemplation Versus Action Stage

The fourth column of Table 4 shows the OR for being in the action stage versus being in the contemplation stage for each variable listed. The odds of being in the action versus the contemplation group decrease the more barriers to preventive action for hearing loss one perceives (OR 0.808, 95% CI 0.680-0.959) and increase the more one has self-efficacy in the prevention of hearing loss (OR 1.431, 95% CI 1.196-1.711).

### Communication Needs and Preferences

More than half of the participants (n=249, 51%) expressed an interest in expanding their knowledge on the topics of hearing loss and safe listening in the context of video gaming and esports. While investigating the preferred channels for obtaining such information, it was found that no single mode was unequivocally favored. Nonetheless, noteworthy options included instruction manuals for gaming devices, dedicated websites, and user interfaces integrated into gaming devices. No significant differences were observed in preferences for sex, age, or education. Regarding trusted sources of information, respondents showed a clear preference for health care professionals and governmental or international agencies (eg, WHO), while traditional media and religious leaders were among the least trusted sources. Female respondents reported significantly higher trust in government or international health agencies (eg, WHO) compared to male respondents (*F*_1,470_=8.76; *P*=.003; η_p_^2^=0.018). Younger respondents reported higher trust in government or international health agencies (*F*_1,486_=4.98; *P*=.03; η_p_^2^=0.010) as well as higher trust in producers of gaming or esports devices (*F*_1,486_=15.12; *P*<.001; η_p_^2^=0.030) and doctors or pharmacists (*F*_1,486_=12.59; *P*<.001; η_p_^2^=0.025). Significant differences in trust were observed across educational levels, with respondents holding lower education reporting higher trust in producers of gaming or esports devices (*F*_2,468_=4.88; *P*=.008; η_p_^2^=0.020) as well as higher trust in newspapers or magazines (*F*_2,468_=4.00; *P*=.02; η_p_^2^=0.017). More details about communication needs and preferences can be found in Table 5.

**Table 5:** Communication needs and preferences (N=488).

		Values
**Interest in receiving more information, n (%)**		
	Yes	249 (51)
**Preferred channels^a^, mean (SD)**		
	Instructions of gaming device	5.91 (1.58)
	User interface of gaming device	5.84 (1.79)
	Dedicated website	5.82 (1.65)
	Social media	5.70 (1.78)
	Mass media	5.43 (1.89)
	Public health campaign	5.29 (1.95)
	Written information	4.90 (2.14)
	Interpersonal communication	4.86 (2.11)
	Public events	4.20 (2.31)
**Trust in sources of information^b^, mean (SD)**		
	Health care professionals	6.22 (1.32)
	Governmental or international agencies	6.01 (1.46)
	Charitable organizations	4.65 (1.76)
	Producers of gaming devices	4.48 (1.90)
	Family or friends	4.21 (1.66)
	Internet	4.19 (1.65)
	Radio	3.87 (1.80)
	Newspapers or magazines	3.85 (1.80)
	Television	3.78 (1.75)
	Religious organizations or leaders	2.74 (1.98)

^a^Scores ranging from 1 to 7, with higher scores indicating a higher willingness to receive information from the source.

^b^Scores ranging from 1 to 7, with higher scores indicating higher trust in the source.

## Discussion

### Overview

In this study, we aimed to explore the gaming and esports behaviors, related listening habits, and awareness about hearing health risks among gamers and esports participants. This investigation was motivated by the increasing concerns over potential hearing damage due to prolonged exposure to high sound levels in video gaming environments and the need for designing and implementing specifically designed features that make video games safer.

### Principal Findings

Our first specific aim was to gain insights into the listening habits and safe listening behaviors of video gamers and esports players, exploring the determinants of these behaviors. Our results show that an important part of players set the volume at high or very high levels, with younger participants reporting significantly higher volume settings than older respondents. Furthermore, we observed that most viewers and players consider sounds in esports to be (very) important, and the volume level for esports participation was higher than for viewing. Although most respondents were aware of the risks of loud sound exposure and recognized the benefits of prevention, they still engaged in risky listening behaviors. For instance, only about one-third of video game and esports players reported taking a break from sound every hour, and only a few reported checking the sound dosage. This lack of protective behavior is particularly concerning, given that around half of the video game and esports players reported having experienced at least once a ringing or feelings of fullness or fuzziness in their ears after playing. These findings suggest that many video gamers and esports players are experiencing symptoms of hearing damage, yet are not taking the necessary steps to protect their hearing.

Second, we aimed to understand the link between attitudes, beliefs, awareness, and stages of change regarding auditory health. Applying the constructs of the HBM, we were able to determine that, despite an overall recognition of the risks associated with loud sound exposure, several factors get in the way of a positive behavior change. For instance, many believed that turning down the volume would limit their enjoyment of video games and esports. In addition, while many rated the perceived severity of hearing loss as high, their perceived susceptibility to it was relatively low. Finally, low self-efficacy also seems to play a role in the decision to adopt safer listening practices. These results are in line with what is suggested by current theories of behavior change and evidence in the field of health behaviors and of safe listening in other contexts, such as findings in physical activity promotion, where individuals are often aware of the benefits of exercise but are deterred by perceived barriers like time constraints or lack of motivation [29]. Similarly, in safe listening research for personal audio devices, users often understand the risk of hearing damage but continue to listen at high volumes, believing that lowering the volume would reduce sound quality or enjoyment, especially in noisy environments [13]. Concretely, these findings suggest a need for education programs specifically designed to show how prevention does not require particular skills and does not necessarily limit enjoyment (eg, through testimonials [30]) and to increase the perceived susceptibility [31] to hearing loss among video gamers and esports players.

We also observed a balanced distribution of participants into the 3 readiness-to-change groups, as defined by the TTM [24], with most of them belonging to the “contemplation” stage, followed by the “action” stage and the “precontemplation” stage. While many video gamers and esports players are aware of the risks of loud sound exposure and are considering taking action, they may need additional support to move from contemplation to action. The multivariate analysis revealed several factors that were associated with participants’ readiness to change their listening behavior in the context of video gaming and esports. Participants who perceived themselves as more susceptible to hearing loss and recognized the benefits of preventive actions were less likely to be in the precontemplation stage and more likely to be in the contemplation stage. On the other hand, participants who perceived hearing loss as severe and had more self-efficacy in preventing hearing loss were more likely to be in the precontemplation stage. Participants who had more self-efficacy in preventing hearing loss were more likely to be in the action stage than in the contemplation stage. Finally, participants who perceived more barriers to preventive actions were less likely to be in the action stage. These findings suggest that interventions aimed at promoting safe listening habits among video gamers and esports viewers and players should be tailored to participants’ beliefs and attitudes about hearing loss and preventive actions [32]. In particular, our findings suggest that interventions to increase self-efficacy [33] may be effective in promoting safe listening habits among video gamers and esports players independent of the stage they are in. At the same time, interventions for those in the precontemplation stage should focus on increasing the perceived susceptibility and on showing the benefits of prevention, while those targeted at those in the contemplation stage should include information aimed at reducing barriers, for instance, by showing how listening to lower volumes does not reduce enjoyment.

Finally, we aimed to identify the most effective sources of information that can be leveraged to promote safer listening practices. Approximately half of the participants expressed a keen interest in expanding their knowledge on hearing loss and safe listening in the context of video gaming and esports, suggesting a good degree of openness to information. Regarding trusted sources of information, respondents showed a clear preference for health care professionals and governmental or international agencies. Female respondents showed significantly more trust in governmental or international agencies, while younger respondents showed more trust in health care professionals, governmental or international agencies, and producers of gaming devices. This information is important, as it can inform future communication efforts especially tailored to video gamers and esports players.

Overall, our findings not only highlight the importance of promoting safe listening behaviors among video gamers and esports players but also contribute to the broader literature on behavior change. By identifying key barriers and beliefs unique to this population—such as prioritizing immersion and performance over auditory health—our research offers new insights into how behavior change models, like the HBM, can be tailored to different contexts. This contributes to a more nuanced understanding of how interventions should be tailored to address context-specific motivations and barriers. In doing so, our study advances the development of targeted interventions aimed at reducing unsafe listening habits, with the potential to inform both gaming platform design and broader behavior change strategies. These findings are significant for the ongoing development of health promotion strategies that encourage long-term behavioral change, reinforcing the importance of adapting existing models to the specific needs of the different communities.

### Practice Implications

Our findings support the need for the design and implementation of safe listening features in video games and also provide useful insights into how these features could look like. First, the substantial engagement in video gaming and the pronounced importance of sound highlight the necessity for interventions that do not compromise sound quality. Features enabling the tracking of the sound dose, such as dosimetry [34] or dynamic range compression [35], have emerged as crucial innovations, which could ensure that gamers can remain engaged and fully immersed without risking their hearing health.

Second, the observation of gamers frequently listening at high volumes and not engaging in protective practices, such as regular sound breaks, motivates the introduction of features enabling an automatic reduction of volume [36] and in-game notifications [37] related to measured sound exposure. These interventions would directly encourage gamers to adjust their volume settings and take necessary breaks, promoting safer listening habits organically within the gaming environment.

Third, the reported adverse effects, including ringing in the ears and feelings of fullness, highlight the need for features allowing users to remove particularly harmful sounds [38] and, considering the detrimental impact of tinnitus in terms of psychological or emotional effects, sleep disturbance, auditory issues, and overall health [39], support the introduction of general safe listening warnings in video games. These features aim to provide a more comfortable gaming experience for individuals experiencing tinnitus, reduce the experiences of tinnitus, and raise awareness about the potential adverse effects of unsafe listening practices.

Fourth, despite participants being somewhat knowledgeable about the risks of hearing damage, the application of this knowledge was limited, indicating a gap between awareness and protective action. This gap underscores the importance of user guides, detailing what can be done at different levels, and adaptive headphone safety modes [40,41] that automatically optimize listening settings for safety when switching from a loudspeaker to headphones, directly addressing the need to bridge knowledge with actionable safe listening practices.

Fifth, the significant interest among gamers in expanding their knowledge about hearing health and safe listening practices supports the creation of a safer listening mode. Besides directly contributing to protecting users’ hearing, the very existence of such a mode could educate them on safer listening practices, addressing the community’s desire for information and tools to protect their hearing health.

Finally, our findings show that gamers often face barriers to adopting safer listening practices due to concerns about compromising their gaming experience. This suggests a need for customizable features that balance individual preferences with hearing safety. Features like sound category controls [42,43] and dynamic range tests [44], tailored to specific gaming contexts, could help ensure safe listening without detracting from the immersive experience.

### Limitations

This study has several limitations to consider when interpreting the results. First, relying on self-reported data may introduce bias and may not accurately reflect actual behaviors. Second, the cross-sectional nature of the study prevents causal inferences. Third, the lack of assessment of noise-canceling headphone use, which could mitigate hearing damage, limits the findings. Finally, the open web-based survey recruitment strategy, shared via social media and stakeholder networks, may have led to selection bias, as those more interested in hearing health or gaming were more likely to participate. Despite this, the consistency of our findings with previous studies on related topics [13,14] supports their validity, allowing us to draw meaningful conclusions. Future research should address these limitations and evaluate the effectiveness of educational interventions in promoting safe listening habits among video gamers and esports players.

### Conclusions

This study underscores the urgency of developing interventions to foster safe listening habits in video gamers and esports enthusiasts, pointing out their general lack of awareness and preventive actions against hearing damage. Besides stressing the need for effective communication strategies tailored to this group’s specific preferences, the findings support creating targeted interventions to encourage safe listening and minimize hearing loss risk among these audiences. Incorporating safe listening features directly into video games could offer a strategic method to address prolonged exposure to high sound levels without detracting from the gaming experience. These personalized, gamer-friendly solutions could significantly boost auditory health awareness and practices. The insights into gamer behavior and preferences provided by this study not only enrich discussions on digital health interventions but also pave the way for enhancing hearing health in the digital entertainment sector, contributing to the broader goal of leveraging technology to improve health outcomes.

## Appendix

Multimedia Appendix 1 Survey questionnaire.

Multimedia Appendix 2 Checklist for Reporting Results of Internet E-Surveys (CHERRIES).

## Abbreviations

CHERRIES: Checklist for Reporting Results of Internet E-Surveys
HBM: health belief model
HRA: Human Research Act
LHQ: Listening Habits Questionnaire
OR: odds ratio
TTM: transtheoretical model
WHO: World Health Organization

## Data Availability

The datasets generated and analyzed during this study are available from the corresponding author on reasonable request.
