# Supplementary material for: Safe Listening Beliefs, Attitudes, and Practices Among Gamers and Esports Participants: International Web-Based Survey
Source: JMIR Form Res. 2025 Mar 25;9:e60476. doi: 10.2196/60476 (PMC11979545; doi:10.2196/60476)
Supplement: Multimedia Appendix 1 [file formative_v9i1e60476_app1.pdf]

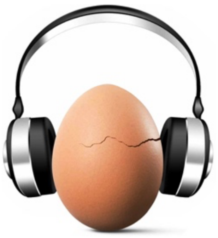

World Health Organization

UNIVERSITÄT LUZERN

FAKULTÄT FÜR GESUNDHEITS-WISSENSCHAFTEN UND MEDIZIN

CENTER FOR REHABILITATION IN GLOBAL HEALTH SYSTEMS

Thank you very much for your interest in participating in this study. The present research is carried out by the Center for Rehabilitation in Global Health System of the University of Lucerne (Lucerne, Switzerland) in collaboration with the World Health Organization (Geneva, Switzerland).

The main objective of the study is to explore people's listening habits when engaging in gaming and e-sports.

**Please fill out the questionnaire only if you are a gamer or if you watch and/or practice e-sports!**

The questionnaire consists of three main sections. The first section includes questions about your listening habits when gaming or when engaging in e-sports. The second questions is about your opinion on several aspects of hearing health. The last section is devoted to collecting some socio-demographic information that will be used for analytical purposes.

Completing the questionnaire will require about 15 minutes.

Your participation to this study is completely voluntary and you have the right to withdraw your participation at any time, without giving a reason, by simply closing the window of your browser.

We guarantee that, if the results of the present study will be made public in scientific papers, or in any other way, this will happen in a completely anonymous manner. Your personal data will not be viewed by or handed to third parties without your explicit permission.

Should you have any questions regarding the study or the questionnaire please feel free to contact Nicola Diviani, Center for Rehabilitation in Global Health System, University of Lucerne at nicola.diviani@unilu.ch.

( ) Please click here to confirm that you have read carefully and understood all the information on this page and that you agree to participate

## Block 0 – Socio-demographics

To start, we would like you to respond to a few questions about yourself. Your answers will be useful for descriptive and statistical purposes. Please remember that all your data will be handled and analyzed in an anonymous and confidential manner.

**A. What is your gender?**
( ) Male
( ) Female
( ) Other
( ) I prefer not to say

**B. What is your year of birth?**

| |
|---|
| |

**C. In which country do you currently reside?**

| |
|---|
| |

**D. What is the highest level of education you have completed?**
( ) Less than High School
( ) High School / GED
( ) Some College
( ) 2-year College Degree
( ) 4-year College Degree
( ) Master’s Degree
( ) Doctoral Degree
( ) Professional Degree (JD, MD)
( ) I prefer not to say

## Block 1 – Listening habits

### 1a Gaming

**Q1. How often do you play video games (hours per week)?**
( ) Never > *go to Q14*
( ) 0-1 hours
( ) 1-2 hours
( ) 3-4 hours
( ) 4-6 hours
( ) 6-10 hours
( ) 10 – 15 hours
( ) 15 – 30 hours
( ) 30+ hours

**Q2. How do you play video games?**
*Please check all that apply.*
( ) Mobile
( ) Stationary console (e.g., Xbox, Playstation, Nintendo)
( ) Hand-held device
( ) PC / Mac
( ) Other, please specify: ____________________________

**Q3. What type(s) of games do you like to play?**

*Please check all that apply.*
( ) Adventure
( ) Board or Card
( ) Shooter
( ) Sports or Simulation
( ) Action
( ) Role-playing
( ) Battle Arena
( ) Survival or Horror
( ) Real-time Strategy
( ) Platformer
( ) Other, please specify: ___________________________

**Q4. On days you listen with your earphones/headphones, how many hours do you usually use them in total for the day?**
( ) Less than half an hour
( ) Half an hour to 1 hour
( ) 1 to 2 hour(s)
( ) 3 to 4 hours
( ) More than 4 hours

**Q5. How important are sounds in a video game for you**
( ) 1 Not at all
…
( ) 5 Very important

**Q6. Why?**

| |
|---|
| |

**Q7. Through what means do you listen to video game sounds?**
*Please check all that apply.*
( ) Headphones
( ) Earphones
( ) External Speakers
( ) Internal Device Speakers

**Q8. When you play video games, what volume setting do you usually listen at?**
( ) 1 Lowest volume
…
( ) 10 Maximum volume

**Q9. How would you describe your typical listening volume when playing video games?**
( ) As loud as a quiet library (40 decibel)
( ) As loud as a conversational speech (60 decibel)
( ) As loud as a vacuum cleaner (80 decibel)
( ) As loud as a blender (85 decibel)
( ) As loud as a lawnmower (90 decibel)
( ) As loud as a chainsaw (100 decibel)

**Q10. Do you take a break from sound every hour?**
( ) Yes
( ) No

**Q11. Do you check information provided through your gaming device on how much time you spend listening, and how high your sound dosages are?**

( ) Yes
( ) No > *go to Q12*
( ) I don’t know if my gaming device has this feature > *go to Q12*
( ) My gaming device does not have this feature > *go to Q12*

**Q11a. If yes, does this information change your playing and listening behaviours?**
( ) Yes
( ) No

**Q12. How often do your ears ring after playing video games?**
( ) Never
( ) Rarely
( ) Sometimes
( ) Frequently
( ) Always

**Q13. How often do your ears feel full or fuzzy after playing video games?**
( ) Never
( ) Rarely
( ) Sometimes
( ) Frequently
( ) Always

1b e-sports

**Q14. Do you view or participate in e-sports events?**
( ) View > *go to Q15*
( ) Participate > *go to Q22*
( ) Both > *go to Q15*
( ) Neither > *go to Q31*

**The following questions refer to when you watch e-sports events.**

**Q15. How do you view e-sports events?**
( ) In-person
( ) Online
( ) Both in-person and online

**Q16. How often do you view e-sports events?**
( ) Daily
( ) Weekly
( ) Monthly
( ) Yearly
( ) Less than yearly

**Q17. How long do you spend viewing e-sports**
( ) 1 hour per day
( ) 1-2 hours per day
( ) 2-4 hours per day
( ) 4-8 hours per day
( ) 8+ hours per day

**Q18. When you watch an eSport event, what volume setting do you usually listen at?**
( ) 1 Lowest volume
…

( ) 10 Maximum volume

**Q19. How would you describe your typical listening volume when you watch an eSport event?**
( ) As loud as a quiet library (40 decibel)
( ) As loud as a conversational speech (60 decibel)
( ) As loud as a vacuum cleaner (80 decibel)
( ) As loud as a blender (85 decibel)
( ) As loud as a lawnmower (90 decibel)
( ) As loud as a chainsaw (100 decibel)

**Q20. How often do your ears ring after you watch an e-sports event?**
( ) Never
( ) Rarely
( ) Sometimes
( ) Frequently
( ) Always

**Q21. How often do your ears feel full or fuzzy after you watch an e-sports event?**
( ) Never
( ) Rarely
( ) Sometimes
( ) Frequently
( ) Always

**The following questions refer to when you participate in e-sports events (including training).**

**Q22. How often do you participate in e-sports (including training sessions)?**
( ) Daily
( ) Weekly
( ) Monthly
( ) Yearly
( ) Less than yearly

**Q22. How long do you spend participating in e-sports events?**
( ) 1 hour per day
( ) 1-2 hours per day
( ) 2-4 hours per day
( ) 4-8 hours per day
( ) 8+ hours per day

**Q23. When you participate in an eSport event, what volume setting do you usually listen at?**
( ) 1 Lowest volume
…
( ) 10 Maximum volume

**Q24. How would you describe your typical listening volume when you participate in an eSport event?**
( ) As loud as a quiet library (40 decibel)
( ) As loud as a conversational speech (60 decibel)
( ) As loud as a vacuum cleaner (80 decibel)
( ) As loud as a blender (85 decibel)
( ) As loud as a lawnmower (90 decibel)
( ) As loud as a chainsaw (100 decibel)

**Q25. How often do your ears ring after you participate in an e-sports event?**
( ) Never
( ) Rarely
( ) Sometimes
( ) Frequently
( ) Always

**Q26. How often do your ears feel full or fuzzy after you participate in an e-sports event?**
( ) Never
( ) Rarely
( ) Sometimes
( ) Frequently
( ) Always

**The following questions refer to when you either watch or participate in e-sports events.**

**Q27. How important are sounds in an eSport event for you?**
( ) 1 Not at all
…
( ) 5 Very important

**Q27a. Why?**
(Open question)

**Q28. Through what means do you listen to e-sports sounds?**
*Please check all that apply.*
( ) Headphones
( ) Earphones
( ) External Speakers
( ) Internal Device Speakers

**Q29. Do you take a break from sound every hour?**
( ) Yes
( ) No

**Q30. Do you check information provided through your device on how much time you spend listening, and how high your sound dosages are?**
( ) Yes
( ) No > *go to Q31*
( ) I don't know if my device has this feature > *go to Q31*
( ) My device does not have this feature > *go to Q31*

**Q30a. If yes, does this information change your playing and listening behaviours?**
( ) Yes
( ) No

## Block 2 – Knowledge

Below you can find some statements about hearing and hearing loss. Please indicate for each statement if you think it is true or false.

*If you do not know, please do not try to guess but chose the "I don't know" option.*

**Q31. Listening to sounds above 85 dB (Decibel) over a period of time can cause permanent damage to your hearing.**
( ) Definitely false
( ) Probably false
( ) Probably true
( ) Definitely true
( ) I don’t know

**Q32. The amount of time you listen to a sound affects how much damage it will cause.**
( ) Definitely false
( ) Probably false
( ) Probably true
( ) Definitely true
( ) I don’t know

**Q33. Listening to video games/e-sports sounds at loud listening levels may damage hearing.**
( ) Definitely false
( ) Probably false
( ) Probably true
( ) Definitely true
( ) I don’t know

| Block 3 – Stage of change |
| --- |

**Q34. Please indicate to what extent you agree with each of the following statements. When answering, make sure to think specifically to the sounds you listen to when you are gaming or watching/participating in e-sports.**

1 = Strongly disagree; 5 = Strongly agree

a. I don't think my listening habits are problematic.
b. I am trying to listen to sounds at a lower volume and for a shorter time than I used to.
c. I enjoy listening to sounds, but sometimes I do it for too long or at a too high volume.
d. Sometimes I think I should listen to sounds at a lower volume and for a shorter time.
e. It's a waste of time thinking about my listening habits.
f. I have just recently changed my listening habits.
g. It is easy to talk about wanting to lower the volume and shorten the listening times, but I am actually doing something about it.
h. I am at the stage where I should think about listening to sounds at a lower volume and for a shorter.
i. My listening habits are a problem sometimes.
j. There is no need for me to think about changing my listening habits.
k. I am actually changing my listening habits right now.
l. Listening to sound at a lower volume and for a shorter time would be pointless for me.

| Block 4 – HBM Susceptibility |
| --- |

**Q35a. How susceptible to hearing loss do you feel?**
1 = Not at all susceptible; 7 = Very susceptible

**Q35b. What is the chance that you will experience hearing loss from listening to loud sounds?**
1 = Very low chance; 7 = Very high chance

**Q35c. How likely do you think it is that you will experience hearing loss resulting from listening to loud sounds when gaming or when you watch/participate in an eSport event?**
1 = Very unlikely; 7 = Very likely

**Q35d. Would you say that you are the type of person who is likely to experience hearing loss?**
1 = Definitely no; 7 = Definitely yes

| Block 5 – HBM Severity |
|---|

**Q36a. How disruptive would hearing loss be to your quality of life?**
1 = Not at all disruptive; 7 = Extremely disruptive

**Q36b. How disruptive would the cost of treating hearing loss be?**
1 = Not at all disruptive; 7 = Extremely disruptive

**Q36c. How disruptive would it be to have to wear a hearing aid?**
1 = Not at all disruptive; 7 = Extremely disruptive

**Q36d. How disruptive would hearing loss be to your ability to communicate with your friends and loved ones?**
1 = Not at all disruptive; 7 = Extremely disruptive

**Q36e. How disruptive would it be to sustain permanent hearing loss as a result of listening to loud listening to loud sounds when gaming or when you watch/participate in an e-sports event?**
1 = Not at all disruptive; 7 = Extremely disruptive

**Q36d. Overall, how disruptive would hearing loss be in your life?**
1 = Not at all disruptive; 7 = Extremely disruptive

| Block 6 – HBM Perceived benefits of prevention |
|---|

**Q37. Please indicate to what extent you agree with each of the following statements.**
1 = Strongly disagree; 7 = Strongly agree

a. Making sure I listen to sounds at safe levels would prevent me from experiencing hearing loss.
b. Turning my video games/e-sports sounds down to a safe level would be a good thing for me to do.
c. Making sure my video games/e-sports sounds are at a safe level would prevent hearing loss.
d. Setting my volume limiter at a safe level would be a good thing for me to do.
e. Using special earphones that block out background noise when I play video games or watch/participate in e-sports would be a good thing for me to do.

| Block 8 – HBM Barriers |
|---|

**Q38. Please indicate to what extent you agree with each of the following statements.**
1 = Strongly disagree; 7 = Strongly agree

a. If I turned my sounds down to a safe level in a loud environment, I wouldn’t be able to hear them.

b. If I turned my sounds down to a safe level in a loud environment, I wouldn’t enjoy video games/e-sports as much.
c. I don’t know what level my sounds should be turned down to in order to protect my hearing.

Block 9 – HBM Self-efficacy

**Q39. Please indicate to what extent you agree with each of the following statements.**
1 = Strongly disagree; 7 = Strongly agree

a. I feel confident in my ability to monitor the volume at which I listen to video games/e-sports sounds.
b. I feel confident in my ability to make sure I listen to games/e-sports sounds at a safe level.
c. I feel confident in my ability to set the volume limiter of my gaming/e-sports device to a safe level.

Block 10 – HBM Intention

**Q40. Please indicate to what extent you agree with the following statement.**
1 = Strongly disagree; 7 = Strongly agree

- If I knew I was listening at an unsafe level, I would be willing to turn down the volume.

Block 11 – Information-seeking

**Q41. Would you like more information about safe listening and ways to prevent hearing loss?**
( ) Yes
( ) No > *go to Q43*

**Q42. How would you like to receive information about safe listening and ways to prevent hearing loss?**
1 = Definitely no; 7 = Definitely yes

a. Public health campaign (e.g., billboards)
b. Written information material (e.g., leaflet or brochure)
c. Dedicated website
d. User interface of listening device
e. Public event
f. Instructions of gaming/e-sports device
g. Mass media
h. Interpersonal communication
i. Social media (e.g., Facebook or Twitter)

**Q43. In general, how much would you trust information about safe listening from each of the following:**
1 = Not at all; 7 = Completely

a. Doctor or pharmacist
b. Family or friends
c. Newspapers or magazines
d. Radio
e. Internet
f. Television

g. Government or international health agencies (e.g., WHO)
h. Charitable organizations
i. Religious organizations and leaders
j. Producers of gaming/e-sports devices
